# Understanding the Role of Persistent Organic Pollutants and Stress in the Association between Proximity to the World Trade Center Disaster and Birth Outcomes

**DOI:** 10.3390/ijerph19042008

**Published:** 2022-02-11

**Authors:** Miranda J. Spratlen, Frederica P. Perera, Andreas Sjodin, Yuyan Wang, Julie B. Herbstman, Leonardo Trasande

**Affiliations:** 1 Columbia Center for Children’s Environmental Health, Department of Environmental Health Sciences, Mailman School of Public Health, Columbia University, New York, NY 10032, USA; fpp1@cumc.columbia.edu (F.P.P.); jh2678@cumc.columbia.edu (J.B.H.); 2 National Center for Environmental Health, Centers for Disease Control and Prevention, Division of Laboratory Sciences, Atlanta, GA 30341, USA; zrq4@cdc.gov; 3 Department of Population Health, New York University Langone Health, New York, NY 10016, USA; yuyan.wang@nyulangone.org (Y.W.); leonardo.trasande@nyulangone.org (L.T.); 4 Department of Pediatrics, School of Medicine, New York University, New York, NY 10016, USA; 5 Department of Environmental Medicine, School of Medicine, New York University, New York, NY 10016, USA

**Keywords:** World Trade Center disaster, stress, birth outcomes, persistent organic pollutants, prenatal exposure

## Abstract

Fetal growth is affected by exposure to both prenatal stress and environmental contaminants. The attacks on the World Trade Center (WTC) resulted in exposure to chemicals and psychological stress amongst New York City residents. We measured prenatal maternal stress and exposure to persistent organic pollutants (polybrominated diphenyl ethers, polychlorinated biphenyls, and polychlorinated dibenzo-p-dioxins (PCDDs)) in 108 participants from a Columbia University WTC birth cohort. Principal component (PC) analyses were conducted to characterize the mixture of exposure to the three groups of chemicals. We evaluated the associations between geographical exposures (proximity to the WTC disaster) and both chemical exposures (PCs) and stress (demoralization). We then evaluated the effect these exposures (PCs and stress) had on previously reported associations between geographical WTC exposure and birth outcomes (birth weight and birth length) in this study population to understand their individual roles in the observed associations. Geographical exposure via proximity to the WTC was associated with the PC reflecting higher PCDD exposure (PC3) (β = 0.60, 95% CI: 0.03, 1.18 for living/working within 2 miles of the WTC; and β = 0.73, 95% CI = 0.08, 1.38 for living within 2 miles of WTC). Previously reported reductions in birth weight and length associated with WTC proximity (β = −215.2, 95% CI: −416.2, −14.3 and β = −1.47, 95% CI: −2.6, −0.34, respectively) were attenuated and no longer significant for birth weight (β = −156.4, 95% CI: −358.2, 45.4) after adjusting for PC3, suggesting that PCDDs may act as partial mediators in this previously observed association. The results of this study can help focus future research on the long-term health effects of these prenatally exposed populations.

## 1. Introduction

Fetal growth has been shown to be influenced by both maternal stress [1,2,3,4] and exposure to environmental contaminants, including persistent organic pollutants [5,6,7,8,9,10]. In the event of manmade or natural disasters, pregnant women may be exposed to high levels of both of these stressors at once, and they are, therefore, a particularly vulnerable population during these events. The terrorist attacks on the World Trade Center (WTC) on 11, September 2001, were a catastrophic disaster and resulted in the release of thousands of tons of chemicals [11] as well as long-lasting psychological stress amongst both responders [12,13,14] and New York City residents [15]. Exposure to pollutants continued long after the initial collapse due to building rubble fires that burned intermittently for more than three months, in addition to the infiltration of contaminated dust into residents’ homes and ventilation systems [11]. Sampling of dust and ash-laden runoff near the WTC site identified numerous toxic chemicals associated with the disaster including persistent organic pollutants that have been associated with fetal growth, such as polychlorinated biphenyls (PCBs), polybrominated diphenyl ethers (PBDEs), polychlorinated dibenzo-p-dioxins (PCDDs), and polychlorinated dibenzofurans (PCDFs) [16,17,18,19]. Biomonitoring studies also identified elevated PCDD/F and perfluorinated compound (PFAS) levels among firefighters [20] and New York State employees and National Guard personnel [21,22] who responded to the disaster. Less is known in regard to the exposures of pregnant women residing near the WTC disaster. Previous studies in the same NYC birth cohort reported associations between geographic proximity to the WTC disaster and exposure to both PFAS [23] and polycyclic aromatic hydrocarbons (PAH)-adducts [24] among pregnant women; however, a separate birth cohort using a different metric to categorize “exposed” pregnant women, found no differences in PCBs, PBDEs, or PCDD/Fs between high- and low-exposure groups [25].

Post-traumatic stress has been well documented in WTC recovery workers [12,14,26], and increased utilization of mental health services has been reported in the general Manhattan population [15] following the attacks. Maternal stress during pregnancy [3,4], and even before conception [27], including disaster-related stress, has been consistently associated with adverse birth outcomes. Indeed, among pregnant women residing in close proximity to the WTC disaster, post-traumatic stress symptomatology [28] and probable WTC-related post-traumatic stress disorder [29] have been associated with decrements in fetal growth. Impacts of WTC-related stress on fetal growth were even identified in non-NYC populations, including Arab populations in California [30] and a Dutch population in the Netherlands [31]. Several other studies have reported associations between different metrics of WTC exposure (e.g., geographic proximity, working at the WTC site, experiencing injuries from the disaster) and adverse birth outcomes, including intrauterine growth restriction [32], low birth weight [29,33], and reductions in birth weight and length [34]. These studies have suggested both stress and environmental contaminants as potential mediators in the observed associations. However, to date, no studies have attempted to measure their roles in the relationship between WTC-related geographic exposure and adverse birth outcomes. In this study, we measured psychological distress in women who were pregnant during the WTC disaster and delivered at hospitals in close proximity to the WTC site, as well as prenatal exposures to various POPs, including PCBs, PBDEs, and PCDD/Fs. We then evaluated the potential mediating effect these variables had on previously reported associations between geographic proximity to the WTC and birth weight and birth length in this study population (Supplemental Figure S1). To our knowledge, this is the first study to try to disentangle the effects of stress and chemical exposures on birth outcomes among pregnant women exposed to the WTC disaster. This is also the first study to evaluate WTC-related exposure to environmental contaminants using mixture analyses.

## 2. Materials and Methods

### 2.1. Study Population

Data for this work came from a Columbia University birth cohort designed to study the effects of WTC exposures on pregnancy outcomes and development. Detailed methods have been described previously [34]. Singleton pregnant women were approached for enrollment from three large downtown hospitals with maternity units, which were selected based on their close proximity to the WTC site, as well as the characteristics of their catchment areas. The three hospitals were Beth Israel and St. Vincent’s (approximately 2 miles from the WTC site) and New York University Downtown (within a half mile of the WTC site). Eligible women were approached for enrollment at the time of labor and delivery, at each of the three hospitals, between 13 December 2001 and 26 June 2002. Eligibility requirements included the following: age between 18 and 39 years; no smoking (>1 cigarette/at any time) during pregnancy; and self-report of no pre-existing diabetes, hypertension, HIV infection, or AIDS and no use of illegal drugs in the last year. Of the 738 women who were initially screened, 369 were eligible and gave consent for participation. Of the 369 eligible women, 329 met full enrollment criteria: (1) contributed at least one blood sample (cord or maternal blood), (2) provided access to their medical record, and (3) completed a 30 to 45 min postpartum interview (conducted in their preferred or native language: English, Spanish, or Chinese), all of which were required for full enrollment in the study.

### 2.2. Sociodemographic and Exposure Variables

The postpartum interview was administered at the hospital in the woman’s preferred or native language (English, Spanish, or Chinese—the three languages spoken in the hospitals’ catchment area). Information on maternal education, date of birth, race, parity, Medicaid status, home smoking exposure, and residential and work addresses was elicited through the interview. Residential and work addresses (for the 4 weeks starting on and following 9/11) were geocoded at the Center for International Earth Science Information Network of Columbia University’s Earth Institute, using geographic information system (GIS) software from the Environmental Systems Research Institute (Redlands, CA), including ArcGIS 8.3 and the Street Map 2003 extension [34]. Using these data, two WTC-related exposure categories were created: (1) women who lived within 2 miles of the WTC site and (2) women who lived or worked within 2 miles of the WTC site. Two miles was selected to delineate the exposure radius based on previous findings of an association between this exposure group and birth outcomes [34], as well as for consistency with the World Trade Center Health Registry definition of the WTC disaster area, which includes the area of Manhattan south of Houston Street and any block of Brooklyn that is within a 1.5 mile radius of the former World Trade Center site [35]. Maternal pre-pregnancy body mass index (BMI) was calculated using weight in kilograms divided by height in meters squared, both abstracted from participants’ medical chart. In the case of missing height (*n* = 36) or weight (*n* = 49) from the medical record, self-reported information on these variables from the hospital interview were used. Child sex and date of birth were abstracted from the child’s medical record. Gestational age in days was also abstracted from the medical record (if missing (*n* = 15), date of mother’s last menstrual period from interview minus child’s date of birth was used). Gestational age on 9/11, used to determine trimester during the WTC disaster, was calculated by subtracting days since the 9/11 disaster on the child’s date of birth from the child’s gestational age in days at birth. Mothers were classified as being in their first trimester on 9/11 if their child had a gestational age of ≤91 days on 9/11 and in their second or third trimester if their child had a gestational age >91 days. Maternal age at delivery was determined by subtracting the child’s date of birth from the mother’s date of birth. Medical complications during pregnancy (including preeclampsia, placental abruption, hypertension, and gestational diabetes) and birth outcomes (child’s birth weight and length) were abstracted from the medical record or obtained from the maternal interview if the medical record was incomplete. Maternal demoralization was used to determine maternal stress and was measured during the postpartum interview using the Psychiatric Epidemiology Research Instrument Demoralization scale (PERI-D). The PERI-D scale provides a measure of nonspecific psychological distress, with demonstrated reliability across different ethnic groups [36,37,38]. Analyses for this study excluded participants without maternal PCDD/F and either maternal or cord PBDE and PCB measurements (*n* = 188). In addition, we removed from the analyses women who completed less than 36 weeks and 6 days of gestation (due to multifactorial and complex causes of preterm birth) (*n* = 5) or had missing data on maternal age, demoralization, race, education, parity, pre-pregnancy BMI, trimester on 9/11, Medicaid status, pregnancy complications, home smoking exposure, child sex, and child birth weight and length (*n* = 28), resulting in a sample size of 108 participants.

### 2.3. Sample Collection and Chemical Analysis

Blood samples from the umbilical cord were collected at the time of delivery; maternal samples were typically collected on the day after delivery. On average, 30.7 mL blood was collected from the umbilical cord, and 30–35 mL blood was collected from the mother. Blood samples were transported to Columbia University laboratory facilities in Northern Manhattan and processed within hours of collection. The buffy coat, packed red blood cells, and plasma were separated and stored at −70 °C.

#### 2.3.1. Polybrominated Diphenyl Ethers (PBDEs)

Detailed methods regarding the analysis of the plasma samples for PBDEs at the Centers for Disease Control and Prevention have been previously described [39,40]. Briefly, the samples were automatically fortified with 13C-labeled internal standards. The samples were subjected to an initial liquid/liquid extraction with hexane:methyl-tert-butyl ether after denaturation with 1 M HCl and isopropanol [39]. Coextracted lipids were then removed on a silica:silica/sulfuric acid column using the Rapid Trace equipment (Zymark, Hopkinton, MA, USA) for automation. Final determination of the target analytes was performed by gas chromatography–isotope dilution high-resolution mass spectrometry employing an MAT95XP (Thermo Finnigan MAT, Bremen, Germany) instrument [40]. Concentrations of target analytes are reported as nanograms per gram lipid weight (weight of plasma lipids) (ng/g). The plasma lipid concentrations were determined using commercially available test kits from Roche Diagnostics Corp. (Indianapolis, IN, USA) for the quantitative determination of total triglycerides (product no. 011002803-0600) and total cholesterol (product no. 011573303-0600). Final determinations were made on a Hitachi 912 Chemistry Analyzer (Hitachi, Tokyo, Japan). In all, 210 cord blood and 163 maternal plasma samples were analyzed for the following PBDE congeners (by International Union of Pure and Applied Chemistry numbers): 2,2,2′,4,4′-tetraBDE (PBDE-47); 2,2′,3,4,4′-pentaBDE (PBDE-85); 2,2′,4,4′,5-pentaBDE (PBDE-99); 2,2′,4,4′,6-pentaBDE (PBDE-100); 2,2′,4,4′,5,5′-hexaBDE (PBDE-153); 2,2′,4,4′,5,6′-hexaBDE (PBDE-154); 2,2′,3,4,4′,5′,6-heptaBDE (PBDE-183); and 2,2′,4,4′,5,5′-hexaBB (BB-153).

#### 2.3.2. Polychlorinated Biphenyls (PCBs), Polychlorinated Dibenzo-p-Dioxins (PCDDs) and Polychlorinated Dibenzofurans (PCDFs)

PCBs and PCDD/Fs were analyzed at the Centers for Disease Control and Prevention. Detailed methods regarding the analysis of PCBs and PCDD/Fs in blood have been previously described [41,42]. Briefly, the samples were spiked with 13C-labeled internal standards, then extracted with organic solvents that were processed through a five-column cleanup procedure. Final determination of the target analytes was performed by gas chromatography–isotope dilution high-resolution mass spectrometry for PCDD/Fs and by gas chromatography–isotope dilution high- and low-resolution mass spectrometry for PCBs. Concentrations of target analytes are reported as nanograms per gram lipid weight (weight of plasma lipids) (ng/g) for PCBs and picograms per gram lipid weight (pg/g) for PCDD/Fs. Final determinations were made on a Hitachi 912 Chemistry Analyzer (Hitachi, Tokyo, Japan). In all, 210 cord blood and 173 maternal plasma samples were analyzed for 36 PCB congeners and 17 PCDD/F congeners. PCB congeners included PCB 18, PCB 28, PCB 44, PCB 49, PCB 52, PCB 66, PCB 74, PCB 87, PCB 99, PCB 101, PCB 105, PCB 110, PCB 118, PCB 128, PCB 138.158, PCB 146, PCB 149, PCB 151, PCB 153, PCB 156, PCB 157, PCB 167, PCB 170, PCB 172, PCB 177, PCB 178, PCB 180, PCB 183, PCB 187, PCB 189, PCB 194, PCB 195, PCB 196.203, PCB 201, PCB 206, PCB 209. PCDD congeners included 2,3,7,8-tetrachlorodibenzo-p-dioxin (2378D); 1,2,3,7,8-pentachlorodibenzo-p-dioxin (12378D); 1,2,3,4,7,8-hexachlorodibenzo-p-dioxin (123478D); 1,2,3,6,7,8-hexachlorodibenzo-p-dioxin (123678D); 1,2,3,7,8,9-hexachlorodibenzo-p-dioxin (123789D); 1,2,3,4,6,7,8-heptachlorodibenzo-p-dioxin (1234678D); and octachlorodibenzodioxin (OCDD). PCDF congeners included 2,3,7,8-tetrachlorodibenzo-furan (2378F); 1,2,3,7,8-pentachlorodibenzo-furan (12378F); 2,3,4,7,8-pentachlorodibenzo-furan (23478F); 1,2,3,4,7,8-hexachlorodibenzo-furan (123478F); 1,2,3,6,7,8-hexachlorodibenzo-furan (123678F); 2,3,4,6,7,8-hexachlorodibenzo-furan (234678F); 1,2,3,7,8,9-hexaachlorodibenzo-furan (123789F); 1,2,3,4,6,7,8-heptachlorodibenzo-furan (1234678F); 1,2,3,4,7,8,9-heptachlorodibenzo-furan (1234789F); and octachlorodibenzofuran (OCDF).

### 2.4. Statistical Analyses

All statistical analyses were conducted in R software (version 3.5.1; R Project for Statistical Computing). Analyses were restricted to compounds detected in ≥50% of maternal and cord samples for 4 PBDEs (PBDE47, PBDE99 PBDE100, PBB153) and 10 PCBs (PCB44, PCB49, PCB52, PCB66, PCB74, PCB99, PCB118, PCB138.158, PCB153, PCB180) and only maternal samples for 3 PCDDs (123678D, 1234678D, OCDD) because no cord samples met this threshold. No maternal or cord PCDF congeners met this threshold and were, therefore, not included in any analyses. To maximize sample sizes, we used both maternal plasma and cord blood PCB and PBDE concentrations. However, to account for differences in maternal versus cord blood samples, we used separate prediction models developed using all available PCB and PBDE samples (121 PBC and 94 PBDE paired cord blood and maternal plasma samples) to transform maternal PCB and PBDE concentrations in participants with maternal measurements but no cord blood measurements. Therefore, cord measurements for these chemicals included actual cord measurements as well as maternal measurements that were transformed through prediction models to reflect what the cord value would have been for that participant had they had a true cord measurement. Prediction models were developed separately for each congener by regressing log-transformed maternal measurements on log-transformed cord measurements. Details of the transformation models are provided (Supplemental Table S1). We transformed maternal plasma samples to cord samples because a greater number of participants had cord blood samples than maternal plasma samples. Correlations between maternal and cord plasma measurements for both PBDEs and PCBs ranged from 0.32 to 0.85 and all were significant (*p* < 0.01) (Supplemental Figure S2a,b). Only maternal concentrations were used for PCDDs because there were no congeners detected in ≥50% of cord blood PCDD samples. In accord with published practices [43], for all PCB, PBDE, and PCDD congeners included in analyses, samples below the limit of detection were imputed as the lipid adjusted concentration divided by the square root of 2. Maternal, cord and combined cord + transformed maternal-to-cord PCB and PBDE, and just cord and maternal PCDD; percentage detected; and geometric mean (range) are displayed in Supplemental Table S2.

The first aim of our analysis was to evaluate the association between our variables representing geographic exposure to the WTC site (WTC proximity exposure variables) and both maternal stress and maternal exposure to PCBs, PBDEs, and PBDDs, as well as the association between maternal stress and maternal exposure to these chemicals with birth outcomes. The second aim of our analysis was to evaluate the potential mediating role of maternal stress and maternal chemical exposure in the previously reported associations between geographic exposure to the WTC site and birth outcomes (Supplemental Figure S1). Because we were interested in evaluating the three chemical groups as a mixture, we used principal component analyses (PCA) for the 108 participants with data on all chemicals to capture their combined exposures. PCA is a data reduction technique, in which the linear relationships between observed correlated variables are captured into a smaller number of principal components. Each input variable, in this case 10 PCBs, 4 PBDEs, and 3 PCDDs, is given a “factor loading”, reflecting the correlation of each chemical with that component [44]. All chemicals were log-transformed and scaled. We followed the Kaiser criterion [45] and included all principal factors with eigenvalues ≥1.0 [46].

Medians (IQR) and percentages were used to describe sociodemographic and birth outcome variables (birth weight and length) in the study population overall and by each of the geographic WTC proximity exposure variables (“Lived or worked <2 miles from the WTC site” vs. “Did not live or work <2 miles from the WTC site”; and “Just lived <2 miles from the WTC site” vs. “Did not live <2 miles from the WTC site”). Differences between participants across geographic WTC proximity exposure categories were calculated using Mann–Whitney U and chi-square tests for continuous and categorical variables, respectively. Linear regression analyses were conducted to evaluate the association between both geographic WTC exposure variables (“Lived or worked <2 miles from WTC site” and “Just lived <2 miles from WTC site”) and each of the chemical principal component (PC) variables as well as maternal demoralization. Linear regression analyses were also conducted to evaluate the association between chemical PC variables and maternal demoralization with birth outcomes. Model adjustments for these analyses were selected a priori and included maternal age, parity, race, education, pre-pregnancy BMI, family smoking status, trimester on 9/11, and child sex. In addition, linear regression analyses were also conducted to evaluate the association between both geographic WTC exposure variables and birth weight and birth length, before and after adjustment for each chemical PC as well as maternal demoralization. Model adjustments for these analyses were selected a priori to be consistent with previous exposure-birth outcome analyses in this cohort [34] and included: maternal race, age, parity, Medicaid status, pregnancy complications, and child sex. Finally, we dichotomized maternal demoralization at the median (representing high and low demoralization) and included an interaction term between this variable and each chemical PC to evaluate their joint association with birth outcomes. We then reported the *p*-value from a two-sided Wald t-test on the coefficient for the two-way multiplicative term. A threshold of *p* < 0.05 was used to define associations as statistically significant.

Due to the large proportion of missing data, to evaluate whether our complete case analysis was biased, in sensitivity analyses, we used inverse probability weighting in which complete cases were weighted by the inverse of their probability of being a complete case. All study variables except for our principal components were used in our model to predict whether a participant was a complete case. We also conducted sensitivity analyses with imputed missing data using multivariate imputation by chained equations (MICE). All study variables were included in the prediction of our imputation model. All study variables with missing data (chemical PCs, demoralization, birth length, parity, race, Medicaid, BMI, and pregnancy complications) were given imputed values if missing. We set our model iterations to 20 and imputations to 30, which achieved healthy convergence of the imputation model evaluated visually through trace and density plots. Because all missing data were imputed, the sample size for these analyses included the full dataset of 329 participants.

## 3. Results

### 3.1. Chemical and Participant Characteristics

**Figure 1:**
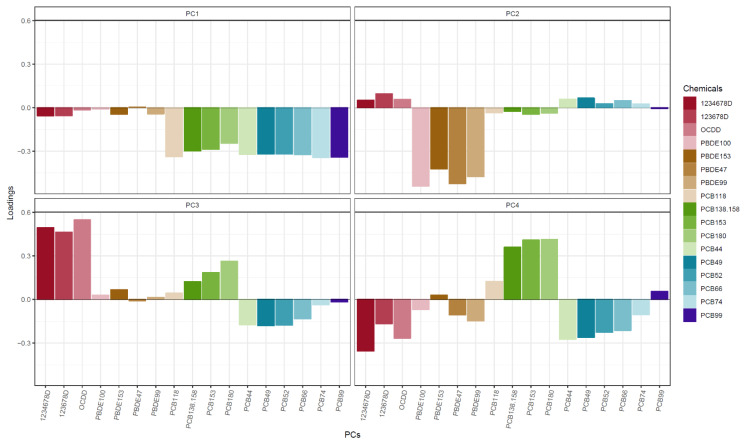
Chemical principal component (PC) loadings.

For all non-supplemental tables and figures, PCB and PBDE concentrations reflect cord + transformed maternal-to-cord measurements, and PCDD concentrations reflect just maternal measurements. A correlation matrix of the 10 PCBs, 4 PBDEs, and 3 PCDDs is displayed in Supplemental Figure S3. PCA showed that the first four PCs captured 85% of chemical exposure variance (data not shown). The first PC (PC1) explained 44% of the variance and was mainly dominated by lower levels of PCBs (Figure 1). The second PC (PC2) explained 19% of the variance and was mainly dominated by lower levels of PBDEs. The third PC explained 13% of the variance and was mainly dominated by higher levels of PCDDs. Finally, the fourth PC explained 9% of the variance and mostly reflected higher PCB 180 and 153, although the loadings for this PC were more spread out across the different chemical congeners.

For the 108 subjects in the analyses, the median maternal age was 31.2 years and median maternal pre-pregnancy BMI was 22 (Table 1). There were slightly more male (52.8%) births than female. The majority of participants were White (53.7%) followed by Asians (23.1%) and Blacks (14.8%). The majority of women had no previous pregnancies (63%), were in their first trimester on 9/11 (61.1%), and were not on Medicaid (69.4%). Most women reported no home smoking exposure (82.4%) and no pregnancy complications (90.7%). Most women had greater than a high school degree (80.6%) with just 11.1% having only a high school degree and 8.3% having no high school degree. The median birth weight was 3441 g and the median birth length was 51 cm. Women who lived or worked farther than 2 miles from the WTC site had higher pre-pregnancy BMI (*p* = 0.004) and were more likely to be on Medicaid (*p* = 0.01).

### 3.2. Associations between Chemical PCs, Demoralization, Geographic WTC Proximity Exposure Group, and Birth Outcomes

**Figure 2:**
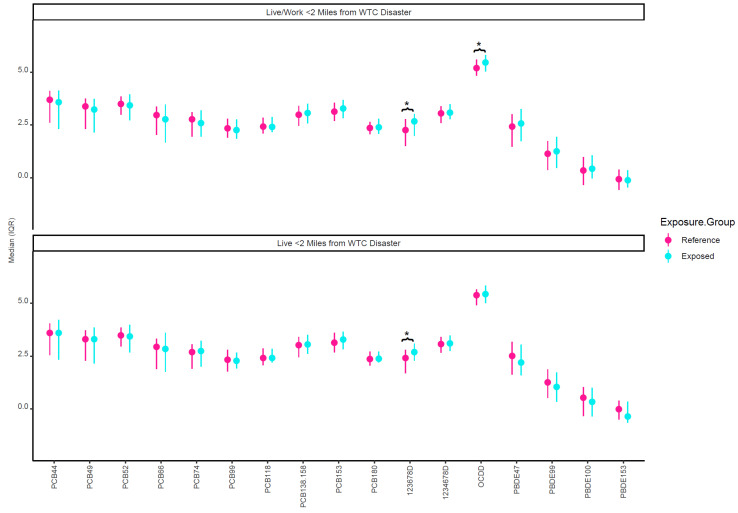
Unadjusted log-transformed lipid-adjusted prenatal chemical concentrations by WTC proximity exposure variables. * Indicates significant difference between exposure groups (*p* < 0.05). The reference group in the top half of the figure represents those who did not live or work within 2 miles of the WTC. The reference group in the bottom half of the figure represents those who did not live within 2 miles of the WTC.

Figure 2 plots the crude median concentrations of the individual chemical congeners by both geographic WTC proximity exposure variables (“living or working within 2 miles of the WTC site” and “just living within 2 miles of the WTC site”). Dioxins 123678D and OCDD were higher in those who lived/worked within 2 miles of the WTC site (exposed group). It was found that 123678D was also higher among those who just lived within 2 miles of the WTC site. In adjusted models, both geographic WTC proximity exposure variables were associated with PC3, the PC reflective of higher maternal PCDD exposures (Table 2). Live or working and just living within 2 miles of the WTC site were associated with 0.60 (95% CI: 0.03, 1.18) and 0.73 (95% CI: 0.08, 1.38) higher PC3, respectively. No other PCs or demoralization were associated with geographic WTC proximity exposure variables. In adjusted models, PC3 was also the only PC associated with birth outcomes (Table 3). Higher PC3 was associated with −96.49 (−163.09, −29.9) g lower birth weight and −0.47 (−0.86, −0.09) cm lower birth length.

**Figure 3:**
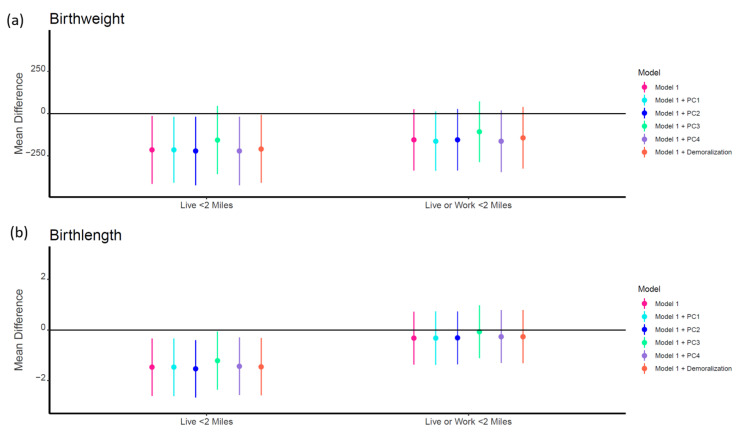
Mean difference (95% CI) in birth outcomes by geographic WTC proximity exposure categories before and after adjustment for chemical principal components (PCs) and maternal demoralization. Model 1 adjusted for maternal race, age, parity, Medicaid status, pregnancy complications, and child sex. The reference group on the left half of the figure represents those who did not live within 2 miles of the WTC. The reference group on the right half of the figure represents those who did not live or work within 2 miles of the WTC.

In adjusted models, exposed groups for both geographic WTC proximity exposure categories were associated with lower birth weight and birth length (Table 4 and Figure 3). However, these associations were only significant for those who lived within 2 miles of the WTC site. Living within 2 miles of the WTC site was associated with −215.2 (95% CI: −416.2, −14.3) g lower birth weight and −1.47 (95% CI: −2.60, −0.34) cm lower birth length. All associations remained consistent after adjustment for PC1 (lower PCBs), PC2 (lower PBDEs), PC4 (higher PCB 180 and 153), and demoralization. However, adjustment for PC3 attenuated all associations, and the relationship between exposure and lower birth weight was no longer significant (β = −156.4, 95% CI: −358.2, 45.4). There were no significant additive interactions between demoralization and PCs; although, there was a significant negative association between PC3 and both birth weight (β = −134.7, 95% CI: −224.0, −45.3) and birth length (β = −0.67 95% CI: −1.18, −0.15) in those with low demoralization but not in those with high demoralization (Supplemental Table S4).

The results from our sensitivity analyses using inverse probability weighting were consistent (Supplemental Tables S5–S7). The results from our sensitivity analyses imputing missing values using MICE were mostly consistent (Supplemental Tables S8–S10). The association between our geographic WTC exposure variables and birth outcomes remained negative; however, the association became significant for living OR working within 2 miles of the WTC and lower birth weight, in addition to the significant negative associations between only living within 2 miles of the WTC and both lower birth weight and lower birth length as seen in the complete case analyses. These associations were all attenuated and no longer significant after adjusting for PC3 (Supplemental Table S10). The associations between geographic WTC exposure variables and PC3 remained significant (Supplemental Table S8). The association between PC3 and birth weight and birth length remained negative; however, the associations were not significant (−43.3, 95% CI: −93.8, 7.08 and −0.27 95% CI: −0.55, 0.01, respectively). Of note, there was a significant positive association observed between PC1 and birth weight (27.2, 95% CI: 1.92, 52.5) (Supplemental Table S9), which was not observed in the complete case analysis.

## 4. Discussion

In this cohort of mother–child dyads who delivered in New York City, NY, in the months following the WTC disaster, we evaluated the mediating role of both chemical exposures and maternal stress in previously observed associations between proximity to the WTC site and lower birth weight and birth length. We used PCA to summarize prenatal exposure to PCBs, PBDEs, and PCDDs and the PERI-D scale (demoralization) to summarize maternal prenatal stress. Four PCs captured most (85%) of the variance in chemical exposures. We found that both of our geographic WTC proximity exposure variables, mothers who lived or worked within 2 miles of the WTC site and mothers who just lived within 2 miles of the WTC site, were associated with the PC reflective of higher exposure to PCDDs. Demoralization was not associated with either geographic WTC proximity exposure variable. We also found that both our geographic WTC proximity exposure variables were associated with lower birth weight and birth length; however, this relationship was only significant for those who lived within 2 miles of the WTC site. After adjustment for each PC as well as demoralization, in separate models, the relationship between proximity to the WTC and birth outcomes remained consistent except when adjusting for the PC reflecting higher PCDD exposure. In this case, the associations were attenuated and no longer significant between living within 2 miles of the WTC site and birth weight, suggesting dioxins may act as partial mediators in these associations. There were no significant additive interactions between demoralization and PCs with birth outcomes.

The attacks on the World Trade Center, and their subsequent collapse, resulted in a toxic mixture of chemical exposures to local populations and psychological stress that impacted populations across the world. The effects of these dual exposures on birth outcomes among mothers who were pregnant during the WTC disaster are incompletely understood. Several studies have tried to evaluate the association between proximity to the WTC, and therefore, likely high exposure to both chemical and psychological stressors; however, results have been inconsistent. Lipkind et al. [47] found no significant differences in birth weight, gestational age, low birth weight, or preterm birth when comparing birth outcomes among pregnant women who were enrolled in the World Trade Center Health Registry versus those who resided in New York City but lived >5 miles from the WTC site. Berkowitz et al. [32] and Lederman et al. [34], however, used closer WTC exposure radius cutoffs, <0.5 miles and <2 miles from the WTC site, respectively, to determine their exposed groups and again compared them to New York City–based populations living farther from those cutoffs. Berkowitz et al. [32] found a twofold higher risk for intrauterine growth restriction in their exposed group compared to their unexposed group, with no differences observed for preterm birth, low birth weight, gestational age, or birth weight. Lederman et al. [34], however, found significant reductions in birth weight and birth length, with no effect on head circumference or gestational age, among their exposed population.

These studies suggest an association between proximity to the WTC site and birth outcomes but do not provide information on the specific exposures driving these associations. Our analysis evaluating the association between chemical PCs and demoralization with proximity to the WTC provides evidence that PCDDs were a more significant exposure source than PBDEs, PCBs, or demoralization in populations living or living/working within 2 miles of the WTC. Further, the attenuation of the previously observed relationship (reported by Lederman et al. [34]) between proximity to the WTC and birth weight and birth length after adjustment for PCDDs suggests that this chemical group may have played a partial mediating role in these associations. The importance of PCDDs as a WTC exposure source is consistent with testing of environmental samples immediately following the disaster, which found elevated PCDD/F concentrations on exterior window surfaces [19] as well as dust, water, sediment, and sewage samples collected in and near the WTC site [18]. These findings were not surprising, given that PCDD/Fs are combustion by-products and the attacks resulted in extensive burning of jet fuel and building materials that lasted for up to three months following the buildings’ collapse [11]. Elevated PCDD/F levels have also been reported among firefighters [20], New York State employees, and National Guard personnel [21] who worked at the WTC site. Further, a recent study found significantly higher levels of PCDD/Fs among WTC Health Registry adolescents over 12 years after the WTC disaster occurred than among non-WTC Heath Registry adolescents, suggesting that exposure to these chemicals, considering their biological half-lives, was quite high at the time of the collapse [48]. In contrast, a study conducted among 100 pregnant women living near or within the WTC site at the time of the attacks found no association between the study’s WTC exposure metric, based on a daily dust exposure index derived through a reconstruction of the post-9/11 WTC plume and maternal plasma PCDDs. Nevertheless, PCDD levels found in this study were higher than nationally reported data in the same time frame [49] and similar to levels reported in the current study. Therefore, the lack of association could be a result of the difficulty in creating accurate WTC exposure indices.

The potential partial mediating role of PCDD concentrations in the previously reported relationship between proximity to the WTC and birth outcomes in this study cohort is supported by experimental and epidemiological evidence. The majority of experimental studies focused on 2,3,7,8-TCDD (TCDD) and reported consistent associations between maternal exposure and reduced birth length and weight [50]. In our study, TCDD was not evaluated because no samples were above the limit of detection. However, PCDDs exert their toxic effects mainly through binding to the aryl-hydrocarbon receptors, which are expressed in many human tissues and may offer a possible mechanism for their association with birth outcomes [50]. Epidemiological studies have been less consistent: no significant reductions in birth weight were found among women highly exposed to TCDD due to an accidental trichlorophenol plant explosion in Seveso, Italy [51]; however, studies evaluating summed PCDD exposure and non-TCDD congeners reported significant reductions in birth weight [7,52,53] and length [54]. Despite this, although adjustment for the PC reflecting higher PCDD exposure attenuated the relationship between WTC exposure and birth outcomes, the inverse relationship between proximity to the WTC and birth weight remained, and in the case of birth length, it remained significant.

Thus, these results, and the minimal effect adjustment for other chemical PCs or demoralization had on the relationship between proximity to the WTC and birth outcomes, suggest that other non-measured chemicals may have played a role or that our study’s demoralization variable did not accurately capture maternal stress in response to such a large disaster. Numerous studies have reported post-traumatic stress (PTS) in WTC recovery workers [12,14,26], and increased utilization of mental health services has been reported in the general Manhattan population [15] following the attacks. In general, women have been shown to be more likely than men to develop PTS after traumatic events, including disasters [55]. Indeed, while PTS prevalence was reported to be higher in New York City than anywhere else in the country months after 9/11, they were highest among women [55,56]. General maternal stress during pregnancy [3,4], and even before conception [27], as well as more acute disaster-related stress [57,58,59,60], has been associated with adverse birth outcomes. In contrast to our findings, studies suggest a consistent trend with WTC-related stress: among pregnant women residing in close proximity to the WTC disaster, post-traumatic stress symptomatology [28] and probable WTC-related post-traumatic stress disorder [29] have been associated with decrements in fetal growth. Some studies even suggest that the magnitude of the disaster resulted in stress-related adverse birth outcomes even outside of New York City [31,33]. Therefore, the lack of association we observed between proximity to the WTC and maternal stress may be a result of the PERI-D scale being designed as a measure of general psychological distress that may not accurately capture disaster-related stress or trauma. Another limitation of this study that should be considered when interpreting findings is that our unexposed comparison group resided in New York City and may have been exposed to WTC-related chemicals and stress. This could have attenuated differences in chemical concentrations and stress between comparison groups and may have contributed to the null findings we observed between proximity to the WTC and demoralization, PBDEs, and PCBs. Finally, the study was limited by a relatively small sample size. However, our consistent sensitivity analysis using MICE to impute missing data suggests our complete case analysis was not biased.

## 5. Conclusions

In conclusion, the first three PCs of our PCA revealed the three main chemical groups under study: PCBs, PBDEs, and PCDDs, respectively. The third PC, reflecting higher PCDD exposure, was associated with both living as well as living or working within 2 miles of the WTC on 9/11 among pregnant women in this study. Further, the previously reported associations between geographic WTC proximity exposure variables and birth outcomes were attenuated after adjustment for PC3, suggesting that PCDD exposure may have played the role of a partial mediator in these relationships. The results of this study suggest PCDDs were an important WTC-related prenatal exposure and can help focus future research on the long-term health effects of these prenatally exposed populations, especially in light of previous evidence suggesting that PCDD concentrations may still be elevated in these groups throughout childhood and into adolescence.

## Figures and Tables

**Table 1:** Study characteristics by geographic WTC proximity exposure categories.

		Live or Work WTC Exposure Variable			Live Only WTC Exposure Variable		
Variable	Overall	>2 Miles	<2 Miles	*p*-Value ^a^	>2 Miles	<2 Miles	*p*-Value ^a^
N (%)	108 (100)	54 (50)	54 (50)		81 (75)	27 (25)	
Birth weight (g), median (IQR):	3441 (3069, 3730)	3583 (3113, 3783)	3399 (2964, 3626)	0.04	3515 (3110, 3775)	3335 (3043, 3488)	0.03
Birth length (cm), median (IQR):	51.0 (49.5, 52.6)	51.0 (49.5, 52.9)	51.0 (49, 52.4)	0.37	51.0 (49.5, 53)	50.0 (48.8, 51.8)	0.05
Child sex, *n* (%):							
Female	51 (47.2)	26 (48.1)	25 (46.3)	>0.99	38 (46.9)	13 (48.1)	>0.99
Male	57 (52.8)	28 (51.9)	29 (53.7)		43 (53.1)	14 (51.9)	
Maternal race, *n* (%):							
Black	16 (14.8)	7 (13)	9 (16.7)	0.33	12 (14.8)	4 (14.8)	0.15
White	58 (53.7)	33 (61.1)	25 (46.3)		48 (59.3)	10 (37)	
Asian	25 (23.1)	9 (16.7)	16 (29.6)		16 (19.8)	9 (33.3)	
Other	9 (8.3)	5 (9.3)	4 (7.4)		5 (6.2)	4 (14.8)	
Parity, *n* (%):							
No previous pregnancies	68 (63)	30 (55.6)	38 (70.4)	0.16	51 (63)	17 (63)	>0.99
1+ previous pregnancies	40 (37)	24 (44.4)	16 (29.6)		30 (37)	10 (37)	
Maternal education, *n* (%):							
<High school degree	9 (8.3)	6 (11.1)	3 (5.6)	0.09	6 (7.4)	3 (11.1)	0.33
High school degree	12 (11.1)	9 (16.7)	3 (5.6)		11 (13.6)	1 (3.7)	
>High school degree	87 (80.6)	39 (72.2)	48 (88.9)		64 (79)	23 (85.2)	
Maternal age, median (IQR):	31.2 (27, 34.4)	30.9 (27, 33.6)	31.3 (28.2, 34.5)	0.61	31.3 (27.2, 34.6)	30.9 (25.2, 33.6)	0.45
Maternal pre-pregnancy BMI, median (IQR):	22 (20.1, 25.3)	23.2 (21, 26.5)	21.3 (19.5, 23.1)	0.004	22.5 (20.2, 25.8)	21.5 (20.1, 24.3)	0.49
Family smoking exposure, *n* (%):							
No	89 (82.4)	47 (87)	42 (77.8)	0.31	70 (86.4)	19 (70.4)	0.11
Yes	19 (17.6)	7 (13)	12 (22.2)		11 (13.6)	8 (29.6)	
Trimester, *n* (%):							
First	66 (61.1)	30 (55.6)	36 (66.7)	0.32	49 (60.5)	17 (63)	>0.99
Second/third	42 (38.9)	24 (44.4)	18 (33.3)		32 (39.5)	10 (37)	
Medicaid, *n* (%):							
No	75 (69.4)	31 (57.4)	44 (81.5)	0.01	55 (67.9)	20 (74.1)	0.72
Yes	33 (30.6)	23 (42.6)	10 (18.5)		26 (32.1)	7 (25.9)	
Pregnancy complications, *n* (%):							
No	98 (90.7)	50 (92.6)	48 (88.9)	0.74	73 (90.1)	25 (92.6)	>0.99
Yes	10 (9.3)	4 (7.4)	6 (11.1)		8 (9.9)	2 (7.4)	

^a^ Differences between participants across geographic WTC proximity exposure categories were calculated using Mann–Whitney U and chi-square tests for continuous and categorical variables, respectively. Abbreviations: body mass index (BMI); interquartile range (IQR).

**Table 2:** Mean difference (95% CI) in chemical principal components (PC) by WTC proximity exposure categories.

	PC1	PC2	PC3	PC4	Demoralization
Live/work <2 miles	−0.05 (−1.12, 1.03)	−0.14 (−0.86, 0.59)	0.60 (0.03, 1.18)	0.26 (−0.26, 0.78)	0.11 (−0.07, 0.3)
Live <2 miles	0.15 (−1.07, 1.37)	0.38 (−0.44, 1.2)	0.73 (0.08, 1.38)	0.16 (−0.43, 0.75)	0.00 (−0.21, 0.22)

Models adjusted for maternal age, parity, race, education, pre-pregnancy BMI, family smoking status, trimester on 9/11, and child sex.

**Table 3:** Mean difference (95% CI) in birth outcomes by chemical principal components (PCs) and demoralization.

	Birth Weight (g)	Birth Length (cm)
PC1	22.5 (−9.49, 54.48)	−0.03 (−0.22, 0.15)
PC2	2.16 (−49.77, 54.1)	0.11 (−0.19, 0.4)
PC3	−96.49 (−163.09, −29.9)	−0.47 (−0.86, −0.09)
PC4	−6.81 (−74.25, 60.62)	−0.15 (−0.54, 0.23)
Demoralization	−91.19 (−284.8, 102.42)	−0.37 (−1.48, 0.74)

Models adjusted for maternal age, parity, race, education, pre-pregnancy BMI, family smoking status, trimester on 9/11, and child sex.

**Table 4:** Mean difference (95% CI) in birth outcomes by geographic WTC proximity exposure categories before and after adjustment for chemical principal components (PCs) and maternal demoralization.

	Model 1	Model 1 + PC1	Model 1 + PC2	Model 1 + PC3	Model 1 + PC4	Model 1 + Demoralization
**Birth Weight**						
Live/work <2 miles	−155.9 (−336.7, 25.0)	−163.0 (−338.2, 12.1)	−154.8 (−336.5, 26.8)	−107.7 (−287.5, 72.1)	−164.3 (−346.5, 17.9)	−143.6 (−326.0, 38.7)
Live <2 miles	−215.2 (−416.2, −14.3)	−214.9 (−409.7, −20.2)	−221.9 (−424.3, −19.4)	−156.4 (−358.2, 45.4)	−221.9 (−423.8, −20.0)	−209.5 (−410.4, −8.63)
**Birth Length**						
Live/work <2 miles	−0.32 (−1.36, 0.72)	−0.32 (−1.37, 0.73)	−0.31 (−1.35, 0.73)	−0.07 (−1.11, 0.97)	−0.26 (−1.3, 0.79)	−0.26 (−1.31, 0.79)
Live <2 miles	−1.47 (−2.6, −0.34)	−1.47 (−2.61, −0.34)	−1.53 (−2.67, −0.4)	−1.21 (−2.36, −0.06)	−1.43 (−2.56, −0.29)	−1.45 (−2.58, −0.32)

Model 1 adjusted for maternal race, age, parity, Medicaid status, pregnancy complications, and child sex.

## Data Availability

The data presented in this study are available on request from the corresponding author. The data are not publicly available to protect the confidentiality of study participants.

## Supplementary Materials

The following supporting information can be downloaded at https://www.mdpi.com/article/10.3390/ijerph19042008/s1: Figure S1: Schematic of study associations evaluated; Figure S2a: Maternal–cord correlations for PBDEs (*n* = 94); Figure S2b: Maternal–cord correlations for PCBs (*n* = 121); Figure S3: Correlation matrix of PCDDs, PBDEs, and PCBs; Table S1: Maternal-to-cord transformations of PCBs and PBDEs; Table S2: Geometric means and limits of detection for chemicals in maternal plasma and cord blood; Table S3: Principal component (PC) loadings for PCDDs, PBDEs, and PCBs; Table S4: Interactions between chemical principal components (PCs) and demoralization and birth outcomes; Table S5: Mean difference (95% CI) in chemical principal components (PCs) by WTC proximity exposure categories using inverse probability stabilized weights; Table S6: Mean difference (95% CI) in birth outcomes by chemical principal components (PCs) and demoralization using inverse probability stabilized weights; Table S7: Mean difference (95% CI) in birth outcomes by geographic WTC proximity exposure categories before and after adjustment for chemical principal components (PCs) and maternal demoralization using inverse probability stabilized weights; Table S8: Mean difference (95% CI) in chemical principal components (PCs) by WTC proximity exposure categories using multiple imputation by chained equations; Table S9: Mean difference (95% CI) in birth outcomes by chemical principal components (PCs) and demoralization using multiple imputation by chained equations; Table S10: Mean difference (95% CI) in birth outcomes by geographic WTC proximity exposure categories before and after adjustment for chemical principal components (PCs) and maternal demoralization using multiple imputation by chained equations.
