# Supplementary material for: Understanding the Role of Persistent Organic Pollutants and Stress in the Association between Proximity to the World Trade Center Disaster and Birth Outcomes
Source: Int J Environ Res Public Health. 2022 Feb 11;19(4):2008. doi: 10.3390/ijerph19042008 (PMC8871981; doi:10.3390/ijerph19042008)
Supplement: Supplementary file 1 [file ijerph-19-02008-s001.zip › ijerph-1577685-supplementary.pdf]

Title: Understanding the Role of Persistent Organic Pollutants and Stress in the Association between Proximity to the World Trade Center Disaster and Birth Outcomes

Authors: Miranda J Spratlen*[a], Frederica P Perera[a], Andreas Sjodin[b], Yuyan Wang[c], Julie Herbstman[a§], Leonardo Trasande[c,d,e §]

Author Affiliations: [a]Columbia Center for Children's Environmental Health, Department of Environmental Health Sciences, Columbia University Mailman School of Public Health, New York, New York; [b]Centers for Disease Control and Prevention; National Center for Environmental Health; Division of Laboratory Sciences, Atlanta, GA; [c]Department of Population Health, New York University Langone Health, New York, NY; [d]Department of Pediatrics, New York University School of Medicine, New York, NY; [e]Department of Environmental Medicine, New York University School of Medicine, New York, NY

[§]Co-last authors

Pages: 10
Figures: 3
Tables: 10

<u>Supporting Information</u>

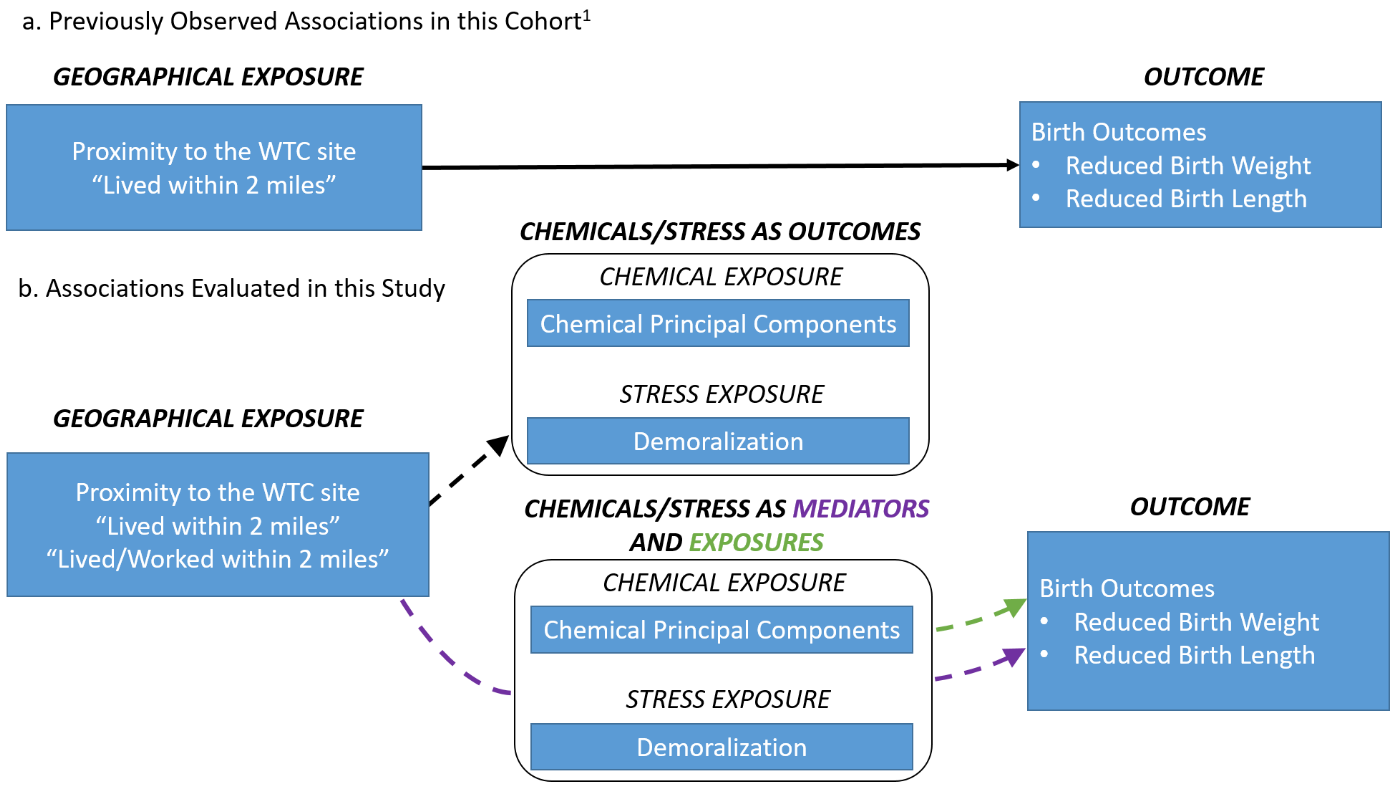

Figure S1. Schematic of Study Associations Evaluated.

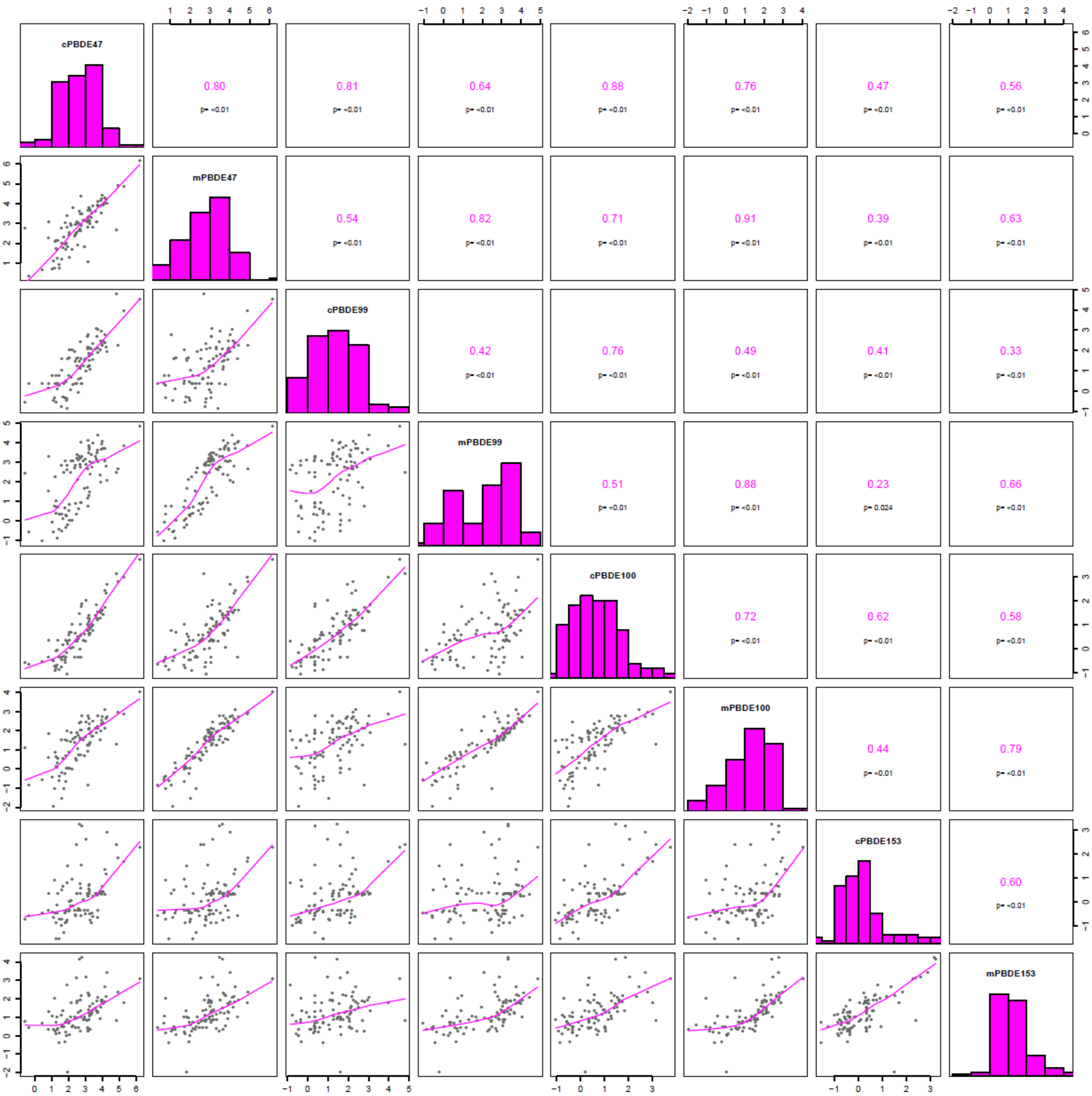

Figure S2a. Maternal – cord correlations for PBDEs (N=94).

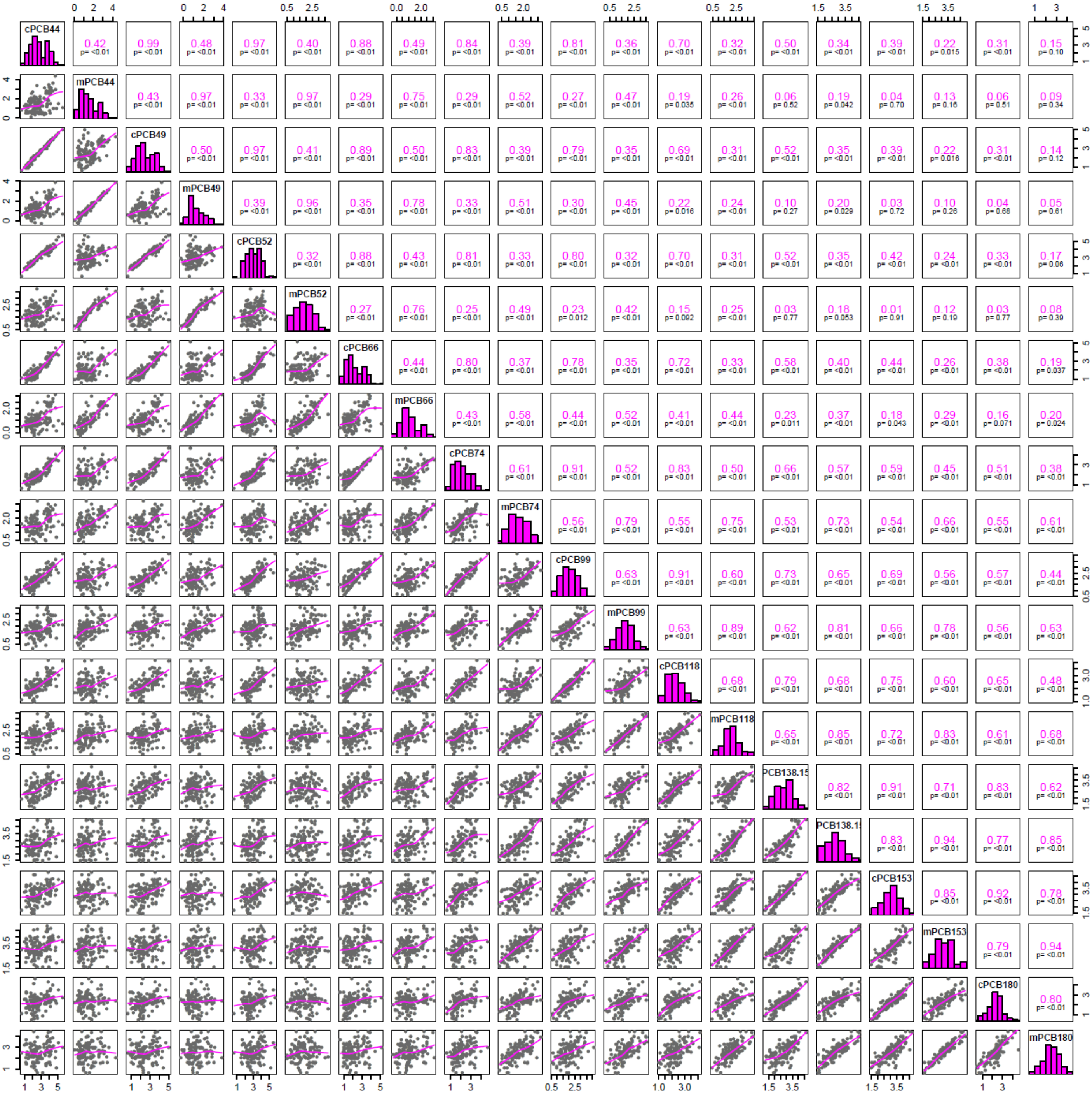

Figure S2b. Maternal – Cord Correlations for PCBs (n=121).

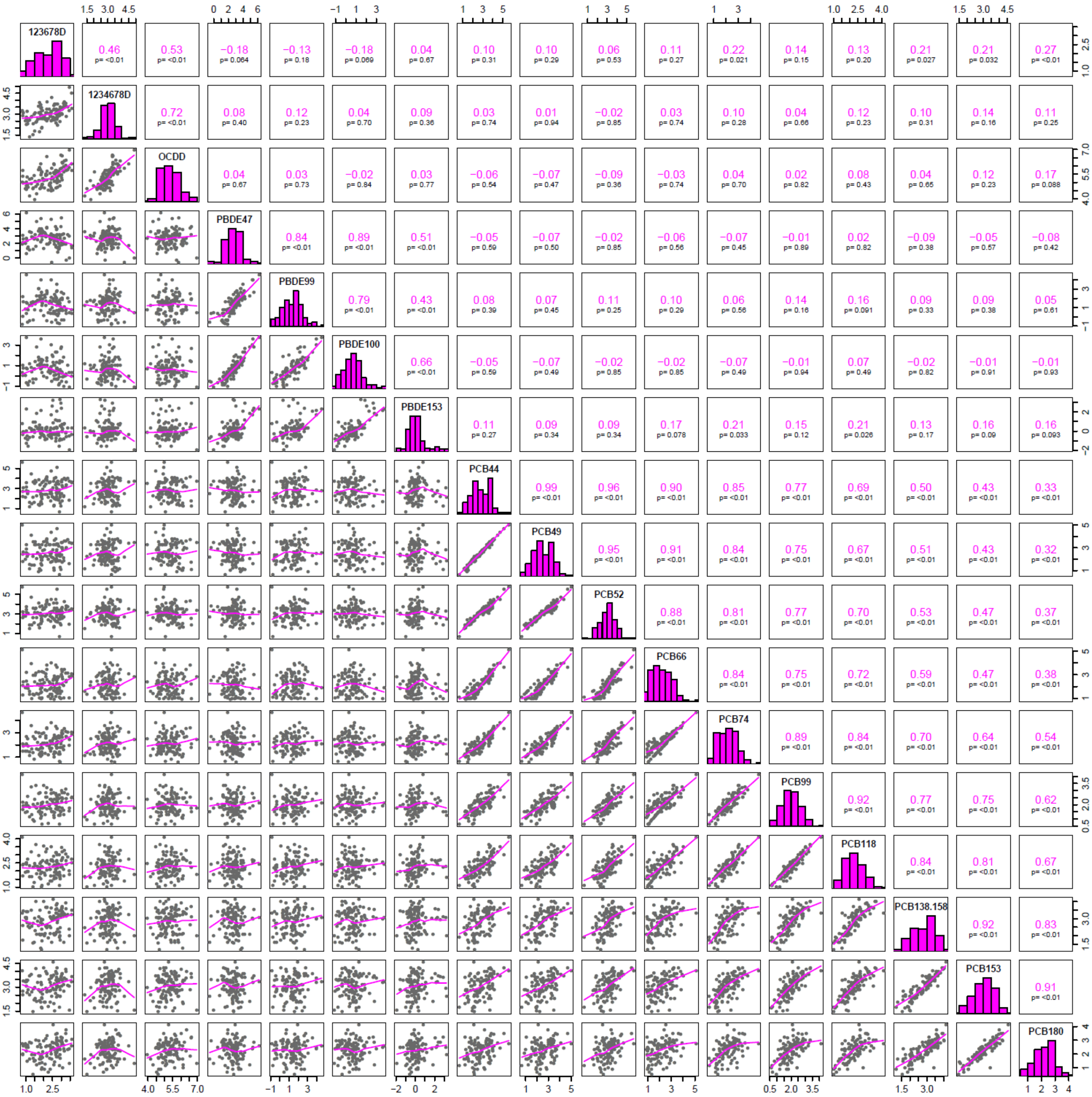

Figure S3. Correlation Matrix of PCDDs, PBDEs and PCBs.

Table S1. Maternal-to-Cord Transformations of PCBs and PBDEs.

Congener	Transformation Formula
PCB44	1.8451133 + (0.6356086*Maternal PCB44)
PCB49	1.6661639 + (0.6929260*Maternal PCB49)
PCB52	2.2510225 + (0.4548271*Maternal PCB52)
PCB66	1.4068923 + (0.7419520*Maternal PCB66)
PCB74	0.8618835 + (0.8145036*Maternal PCB74)
PCB99	0.9187943 + (0.6616968*Maternal PCB99)
PCB118	1.0102781 + (0.6319484*Maternal PCB118)
PCB138.158	0.5663911 + (0.8132915*Maternal PCB138.158)
PCB153	0.5848827 + (0.7930534*Maternal PCB153)
PCB180	0.3893654 + (0.7647683*Maternal PCB180)
PBDE47	0.2534549 + (0.8425674*Maternal PBDE47)
PBDE99	0.5440749 + (0.3799641*Maternal PBDE99)
PBDE100	-0.1280924 + (0.6274888*Maternal PBDE100)
PBDE153	-0.6228235 + (0.6580994*Maternal PBDE153)

Table S2. Geometric Means and Limits of Detection for Chemicals in Maternal Plasma and Cord Blood.

	Cord Blood PCBs (N=210)			*Maternal Plasma PCBs (N=173)*			*Cord and Maternal-to-Cord Transformed (N=251)*	
	**Number Detected (%)**	**Geometric Mean**	**Range**	**Number Detected (%)**	**Geometric Mean**	**Range**	**Geometric Mean**	**Range**
PCB44	191 (91.8%)	27.12	(1.91, 272)	156 (91.8%)	6.49	(1.06, 75.9)	28.21	(1.91, 272)
PCB49	186 (89.4%)	20.12	(1.91, 156)	152 (89.4%)	4.94	(0.78, 46.7)	21.09	(1.91, 156)
PCB52	200 (96.2%)	29.24	(1.91, 303)	168 (98.8%)	7.69	(1.7, 44.8)	29.16	(1.91, 303)
PCB66	134 (64.4%)	14.53	(1.84, 197)	107 (62.9%)	3.87	(0.78, 26.6)	14.87	(1.84, 197)
PCB74	164 (78.8%)	12.89	(1.84, 106)	160 (94.1%)	6.06	(1.41, 26.6)	13.03	(1.84, 106)
PCB99	125 (60.1%)	10.02	(1.84, 61.2)	152 (89.4%)	6.32	(1.2, 34.3)	9.99	(1.84, 61.2)
PCB118	114 (54.8%)	12.01	(2.76, 64.5)	148 (87.1%)	8.29	(1.56, 51.2)	11.86	(2.76, 64.5)
PCB138.158	188 (90.4%)	19.43	(3.3, 106)	169 (99.4%)	16.8	(3.4, 92.3)	19.45	(3.3, 106)
PCB153	192 (92.3%)	23.52	(4.3, 116)	168 (98.8%)	23.15	(3.46, 130)	23.18	(4.3, 116)
PCB180	113 (54.3%)	11.01	(1.63, 101)	155 (91.2%)	12.21	(1.9, 81)	10.71	(1.63, 101)
	Cord Blood PBDEs (N=210)			*Maternal Plasma PBDEs (N=163)*			*Cord and Maternal-to-Cord Transformed (N=264)*	
	**Number Detected (%)**	**Geometric Mean**	**Range**	**Number Detected (%)**	**Geometric Mean**	**Range**	**Geometric Mean**	**Range**
BDE47	176 (83.8%)	11.18	(0.57, 613.1)	156 (95.7%)	17.1	(0.21, 28.9)	11.54	(0.57, 613.1)
BDE99	130 (61.9%)	3.24	(0.28, 202.9)	149 (91.4%)	8.21	(0.71, 507.4)	3.34	(0.28, 202.9)
BDE100	139 (66.5%)	1.66	(0.3, 71.9)	155 (95.1%)	3.67	(0.35, 277.5)	1.7	(0.30, 71.9)
BB153	114 (54.3%)	1.07	(0.21, 28.9)	160 (98.2%)	3.33	(0.14, 106.0)	1.09	(0.15, 28.9)
	Cord Blood PCDDs (N=210)			*Maternal Plasma PCDDs (N=173)*				
	**Number Detected (%)**	**Geometric Mean**	**Range**	**Number Detected (%)**	**Geometric Mean**	**Range**	**NA**	**NA**
123678D	18 (8.6%)	18.61	(5.52, 105.2)	87 (50.3%)	10.5	(1.7, 56.4)	---	---

1234678D	86 (41%)	26.73	(6.08, 245)	153 (88.4%)	21.4	(2.62, 135)	---	---
OCDD	96 (45.7%)	237.01	(56.6, 10100)	145 (83.8%)	204.5	(16.3, 1060)	---	---

Table S3. Principal Component (PC) Loadings for PCDDs, PBDEs and PCBs.

Chemicals	PC1	PC2	PC3	PC4
123678D Maternal	0.05341	0.096757	0.46363	0.166105
1234678D Maternal	0.055326	0.052087	0.494714	0.355394
OCDD Maternal	0.01529	0.058649	0.548409	0.265903
PBDE 47 Transformed	-0.0053	-0.52301	-0.00937	0.105302
PBDE 99 Transformed	0.041776	-0.47427	0.013871	0.14732
PBDE 100 Transformed	0.008802	-0.54024	0.029428	0.070428
PBDE 153 Transformed	0.044896	-0.42086	0.067035	-0.03038
PCB 44 Transformed	0.321525	0.058839	-0.1743	0.274513
PCB 49 Transformed	0.319849	0.066676	-0.18074	0.261297
PCB 52 Transformed	0.319483	0.027815	-0.17725	0.225438
PCB 66 Transformed	0.322968	0.049375	-0.13248	0.21274
PCB 74 Transformed	0.342966	0.027188	-0.03758	0.103459
PCB 99 Transformed	0.341401	-0.00519	-0.01805	-0.05472
PCB 118 Transformed	0.337032	-0.03488	0.042986	-0.12324
PCB 138.158 Transformed	0.298014	-0.02571	0.12194	-0.36034
PCB 153 Transformed	0.285822	-0.04439	0.184492	-0.4091
PCB 180 Transformed	0.244233	-0.03595	0.263448	-0.41342
**Proportion of Variance**	0.44	0.19	0.13	0.09

Table S4. Interactions between Chemical Principal Components (PC) and Demoralization with Birth Outcomes.

	Birth Weight	Birth Length
**PC1**		
Low Demoralization	-45.1 (-92.6, 2.32)	0.13 (-0.15, 0.4)
High Demoralization	-44.9 (-90.2, 0.53)	-0.12 (-0.38, 0.15)
P-Interaction	0.99	0.20
**PC2**		
Low Demoralization	37.7 (-29.9, 105.3)	0.08 (-0.31, 0.47)
High Demoralization	-26.2 (-103.8, 51.3)	0.18 (-0.26, 0.62)
P-Interaction	0.22	0.74
**PC3**		
Low Demoralization	-134.7 (-224.02 -45.3)	-0.67 (-1.18, -0.15)
High Demoralization	-53.6 (-129.5, 22.3)	-0.26 (-0.69, 0.18)
P-Interaction	0.16	0.21
**PC4**		
Low Demoralization	18.6 (-95.4, 132.5)	-0.28 (-0.92, 0.37)
High Demoralization	21.9 (-72.0, 115.8)	-0.22 (-0.75, 0.31)
P-Interaction	0.96	0.89

Models adjusted for maternal race, age, parity, Medicaid status, pregnancy complications, and child sex

Table S5. Mean Difference (95% CI) in Chemical Principal Components (PC) by WTC Proximity Exposure Categories Using Inverse Probability Stabilized Weights.

	PC1	PC2	PC3	PC4	Demoralization
**Live/work <2 miles**	-0.34 (-1.67, 0.98)	-0.36 (-1.03, 0.3)	0.73 (0.24, 1.22)	0.08 (-0.55, 0.71)	0.03 (-0.16, 0.23)
**Live <2 miles**	0.33 (-1.2, 1.87)	0.12 (-0.62, 0.86)	0.76 (0.24, 1.29)	0.3 (-0.45, 1.05)	-0.06 (-0.28, 0.15)

Models adjusted for maternal age, parity, race, education, pre-pregnancy BMI, family smoking status, trimester on 9/11 and child sex

Table S6. Mean Difference (95% CI) in Birth Outcomes by Chemical Principal Components (PCs) and Demoralization Using Inverse Probability Stabilized Weights.

	Birth Weight (g)	Birth Length (cm)
**PC1**	19.3 (-15.2, 53.7)	-0.11 (-0.35, 0.12)
**PC2**	-19.9 (-77.6, 37.8)	0.11 (-0.17, 0.39)
**PC3**	-109.6 (-189.8, -29.5)	-0.57 (-1.06, -0.08)
**PC4**	9.75 (-50.9, 70.4)	-0.17 (-0.66, 0.31)
**Demoralization**	-191.2 (-393.2, 10.8)	-0.60 (-1.71, 0.52)

Models adjusted for maternal age, parity, race, education, pre-pregnancy, BMI, family smoking status, trimester on 9/11 and child sex

Table S7. Mean Difference (95% CI) in Birth Outcomes by Geographic WTC Proximity Exposure Categories Before and After Adjustment for Chemical Principal Components (PC) and Maternal Demoralization Using Inverse Probability Stabilized Weights.

	Model 1	Model 1 + PC1	Model 1 + PC2	Model 1 + PC3	Model 1 + PC4	Model 1 + Demoralization
**Birth Weight**						
Live/work <2 miles	-194.1 (-402.0, 13.7)	-188.2 (-395.8, 19.4)	-203.9 (-410.8, 2.93)	-130.5 (-341.3, 80.3)	-195.3 (-403.9, 13.4)	-187.0 (-390.8, 16.7)
Live <2 miles	-235.1 (-462.2, -8.12)	-242.2 (-482.7, -1.74)	-233.5 (-459.0, -7.99)	-168.1 (-397.3, 61.2)	-239.9 (-464.8, -15.0)	-248.0 (-469.5, -26.5)
**Birth Length**						
Live/work <2 miles	-0.79 (-1.87, 0.30)	-0.83 (-1.93, 0.27)	-0.75 (-1.85, 0.35)	-0.45 (-1.5, 0.6)	-0.77 (-1.85, 0.32)	-0.77 (-1.85, 0.32)
Live <2 miles	-1.81 (-3.23, -0.38)	-1.78 (-3.15, -0.4)	-1.82 (-3.26, -0.39)	-1.5 (-2.8, -0.21)	-1.77 (-3.26, -0.27)	-1.85 (-3.28, -0.43)

Model 1 adjusted for maternal race, age, parity, Medicaid status, pregnancy complications, and child sex

Table S8. Mean Difference (95% CI) in Chemical Principal Components (PC) by WTC Proximity Exposure Categories Using Multiple Imputation by Chained Equations.

	PC1	PC2	PC3	PC4	Demoralization
**Live/work <2 miles**	0.01 (-0.84,0.86)	-0.08 (-0.68,0.52)	0.91 (0.47,1.34)	0.15 (-0.32,0.62)	0.09 (-0.01,0.19)
**Live <2 miles**	0.59 (-0.35,1.53)	0.17 (-0.46,0.79)	0.79 (0.30,1.29)	0.06 (-0.44,0.56)	0.05 (-0.07,0.16)

Models adjusted for maternal age, parity, race, education, pre-pregnancy BMI, family smoking status, trimester on 9/11 and child sex

Table S9. Mean Difference (95% CI) in Birth Outcomes by Chemical Principal Components (PCs) and Demoralization Using Multiple Imputation by Chained Equations.

	Birth Weight (g)	Birth Length (cm)
**PC1**	27.2 (1.92, 52.5)	0.00 (-0.16, 0.16)
**PC2**	6.21 (-34.1, 46.5)	0.20 (-0.06, 0.47)
**PC3**	-43.3 (-93.8, 7.08)	-0.27 (-0.55, 0.01)
**PC4**	27.8 (-21.6, 77.2)	0.00 (-0.33, 0.33)
**Demoralization**	-33.1 (-143.3, 77.1)	-0.28 (-0.95, 0.38)

Models adjusted for maternal age, parity, race, education, pre-pregnancy BMI, family smoking status, trimester on 9/11 and child sex

Table S10. Mean Difference (95% CI) in Birth Outcomes by Geographic WTC Proximity Exposure Categories Before and After Adjustment for Chemical Principal Components (PC) and Maternal Demoralization Using Multiple Imputation by Chained Equations.

	Model 1	Model 1 + PC1	Model 1 + PC2	Model 1 + PC3	Model 1 + PC4	Model 1 + Demoralization
**Birth Weight**						
Live/work <2 miles	-109.2 (-209.6, -8.75)	-109.1 (-209.4, -8.75)	-109.5 (-210.5, -8.47)	-78.7 (-190.8, 33.3)	-113.5 (-214.3, -12.8)	-106.9 (-207.9, -5.91)
Live <2 miles	-140.9 (-252.7, -29.2)	-158.7 (-271.9, -45.6)	-143.1 (-255.4, -30.77)	-114.5 (-234.5, 5.36)	-142.6 (-254.7, -30.6)	-139.5 (-251.5, -27.5)
**Birth Length**						
Live/work <2 miles	-0.31 (-0.92, 0.3)	-0.31 (-0.93, 0.3)	-0.29 (-0.91, 0.33)	-0.07 (-0.75, 0.61)	-0.31 (-0.92, 0.3)	-0.29 (-0.9, 0.32)
Live <2 miles	-0.86 (-1.53, -0.19)	-0.87 (-1.55, -0.19)	-0.90 (-1.58, -0.21)	-0.69 (-1.42, 0.04)	-0.86 (-1.53, -0.19)	-0.85 (-1.52, -0.17)

Model 1 adjusted for maternal race, age, parity, Medicaid status, pregnancy complications, and child sex

Descriptions

Figure S1. Part “a” shows associations between geographical WTC exposure and birth outcomes that have been previously observed and reported in this study population. Part “b” shows associations that were evaluated in the current study.

Figure S2a. The upper panel shows the Spearman pairwise correlation coefficients. The lower panel shows the pairwise scatterplots. The axes indicate values in the log-scale.

Figure S2b. The upper panel shows the Spearman pairwise correlation coefficients. The lower panel shows the pairwise scatterplots. The axes indicate values in the log-scale.

Figure S3. The upper panel shows the Spearman pairwise correlation coefficients. The lower panel shows the pairwise scatterplots. The axes indicate values in the log-scale.

References

1. Lederman SA, Rauh V, Weiss L, et al. The effects of the World Trade Center event on birth outcomes among term deliveries at three lower Manhattan hospitals. Environ Health Perspect. 2004;112(17):1772-1778.
